# Employment of Tartrite of Antimony Externally

**Published:** 1829-10-01

**Authors:** 


### XXIX.

### TARTRITE OF ANTIMONY EXTERNALLY APPLIED. By Dr. FONTANEILLES.

In a paper published in a recent number of our Parisian cotemporary, the REVUE MEDICALE, Dr. Fontaneilles assures us that, for a great many years past, he has employed a solution of tartrite of antimony, in the proportion of one drachm to the pint of warm water, to local inflammations of the skin and subjacent tissues, with very great success. He generally, however, exhibits antimony internally, at the same time, though not in the large doses recommended by Rassori, Tommassini, and others. For instance, he dissolves about three grains of the tartrite in a quart of lemonade or barley water, and gives this to the patient for common drink. It usually produces some nausea, or vomiting, with discharges from the bowels. In erysipelas, he applies the tepid solution, and repeats the application whenever the cloths get dry. In phlegmonous inflammation—in every kind of phlogosis, in short, except carbuncle, he employs the solution, with great advantage. The existence of wounds is no bar to the antimonial, and he takes no pains to prevent it getting into them. When the phlogosis is very intense, Dr. F. does not neglect local or even general depletion as auxiliary to the above plan. He applies this solution to the inflamed breasts of females, and avers that it rapidly dissipates the engorgements of those glands.

But it is not entirely to acute affections of the surface that he confines the antimonial solution. He uses it as a bath, local or general, in cases of pruritus, dartres, and lumbago. In consequence of the homeopathic doctrine of Haneman, our author was induced to try the effects of a twelfth to a twentieth part of a grain every half-hour or every hour, in some diseases; and though he does not consider this plan so efficacious as that of Rassori, especially in acute affections of the head and chest, yet he derived from it very evident advantages in diseases of the abdomen—particularly in fevers, and in what the followers of Broussais denominate gastro-enterites. We think the hint is worth acting upon, in cases of local inflammation, more especially in erysipelas.

